# Dogs (*canis familiaris*) underestimate the quantity of connected items: first demonstration of susceptibility to the connectedness illusion in non-human animals

**DOI:** 10.1038/s41598-021-02791-1

**Published:** 2021-12-02

**Authors:** Miina Lõoke, Lieta Marinelli, Christian Agrillo, Cécile Guérineau, Paolo Mongillo

**Affiliations:** 1 Laboratory of Applied Ethology, Department of Comparative Biomedicine and Food Science, University of Padua, Padua, Italy; 2 Department of General Psychology, University of Padua, Padua, Italy; 3 Padua Neuroscience Centre, University of Padua, Padua, Italy

**Keywords:** Visual system, Animal physiology, Animal behaviour

## Abstract

In humans, numerical estimation is affected by perceptual biases, such as those originating from the spatial arrangement of elements. Different animal species can also make relative quantity judgements. This includes dogs, who have been proposed as a good model for comparative neuroscience. However, dogs do not show the same perceptual biases observed in humans. Thus, the exact perceptual/cognitive mechanisms underlying quantity estimations in dogs and their degree of similarity with humans are still a matter of debate. Here we explored whether dogs are susceptible to the connectedness illusion, an illusion based on the tendency to underestimate the quantity of interconnected items. Dogs were first trained to choose the larger of two food arrays. Then, they were presented with two arrays containing the same quantity of food, of which one had items interconnected by lines. Dogs significantly selected the array with unconnected items, suggesting that, like in humans, connectedness determines underestimation biases, possibly disrupting the perceptual system’s ability to segment the display into discrete objects. The similarity in dogs’ and humans’ susceptibility to the connectedness, but not to other numerical illusions, suggests that different mechanisms are involved in the estimation of quantity of stimuli with different characteristics.

## Introduction

Mathematical abilities are often considered some of the highest cognitive skills of our species. However, studies in cultural^1,2^, cognitive^3,4^ and developmental^5,6^ psychology showed the existence of rudimentary numerical abilities that are independent from educational factors and precede the emergence of language. Such non-symbolic numerical abilities^7–9^ are supposed to be evolutionarily ancient and recent studies suggested they represent the basis of more complex mathematical skills^10,11^. Understanding non-symbolic numerical abilities is hence fundamental to form a broader comprehension of inter-individual differences in mathematical achievements. As in other research fields, developing animal models would help researchers to address issues that cannot be easily investigated in human subjects.

Non-symbolic numerical abilities are widespread among vertebrates. Many species are capable of discriminating larger from smaller sets of objects in different contexts^12–14^. This is not surprising, since such skills are likely to have a direct impact on the individual’s fitness, for example to maximise the food intake or reduce the risk of predation (reviewed in Ref.^15^). Quantity assessments do not rely solely on the overall quantity of the stimulus set. For instance, cumulative surface area—the overall area covered by elements of a visual array—is often used as a proxy for the estimation of numerosity in human e.g.^16^ and non-human animals e.g.^17,18^. Convex hull—the area of a shape that includes all the items of an array—represents another perceptual cue often used to estimate the quantity of items in a set e.g.^19,20^. However, non-quantitative properties can also influence quantity estimation. For example, both humans and rhesus macaques overestimate items in regularly arranged arrays compared to randomly arranged arrays^21^. Furthermore, humans overestimate a large group of centrally located items compared to items located in small groups in the perimeter to form smaller clusters in the solitaire illusion^22–24^, whereas mixed result have been obtained from other species^22,25–27^.

**Figure 1:**
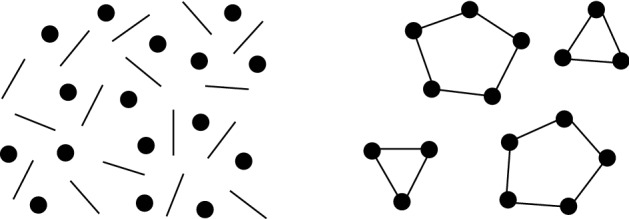
An example of the connectedness illusion. The number of elements connected by lines (right) is typically underestimated compared to unconnected elements (left).

Visual illusions are visual stimuli that are systematically perceived as different from the physical reality^28^. Susceptibility to visual illusions has been studied in a variety of species to shed light into shared mechanisms across taxa^29^. While several visual illusions have been known for millennia, some illusions have been described much more recently. It is the case of the connectedness illusion, a novel numerical illusion that caught the attention of researchers in the last decade^30–32^. The illusion occurs when objects, typically dots, are connected into groups by task-irrelevant lines and such perceptual units are robustly underestimated by the observer (Fig. 1). The underestimation is present already with small set sizes and even if only few dots are connected^31^, although its magnitude increases with more connections^32^, larger group sizes and higher number of dots^30^. The proposed mechanism for the connectedness illusion is the uniform connectedness^33^. Connected objects are perceived as perceptual units, rather than a pair or a group of single objects, and the quantity estimation are based on those perceptual units rather than the veridical numerosity^34^.

Up to date, susceptibility to the connectedness illusion has been assessed only in humans^30–32,34–38^. Given the mixed results of other numerical illusions in non-human animals, such as the solitaire illusion, researchers advanced the hypothesis that perceptual mechanisms associated with quantity estimation in human and non-human animals must be partially dissimilar^22,25–27^. Extending the investigation of the connectedness illusion to non-human animals would help us to deepen this issue and better define similarities and differences between human and non-human animals in the perception of numerosity, also with the respect to the possibility to develop a proper animal model for the study of mathematical abilities in the near future^39^.

In the last two decades, researchers suggested that dogs could be an ideal model for non-invasive comparative neuroscience^40^. In particular, the number of studies investigating dogs’ quantitative abilities have been increasing in recent years^14^. Several studies have shown that dogs are capable of discriminating larger from smaller quantities and their ability is in agreement with the Weber’s law^41–47^. Dogs are able to discriminate up to a ratio of 0.67 spontaneously^47^, and up to 0.80 if trained to do so^45^. Like humans, cumulative surface area can be used by dogs as a proxy of quantity^41^. Only one study to date, assessed if spatial arrangement of objects influences dogs’ quantity perception, using a linear version of the solitaire illusion^47^. However, no misperception of quantity was found by dogs, suggesting that dogs are less susceptible to quantity illusions compared to humans and possibly also to other species^48^.

The aim of this study is to widen the knowledge about the visual perception of quantity by dogs. In particular, our goal is to assess if quantity estimation relies on an unsegmented visual image or a collection of perceptual units created by connecting individual objects. To this, we presented dogs with the connectedness illusion: dogs trained to select the larger of two food arrays were presented with two equally sized panels of food items, one where the elements were connected by task-irrelevant lines and another one with the same amount of isolated elements and lines. If dogs are susceptible to the connectivity illusion as humans^30,35^, we expected them to choose the stimulus with unconnected food elements.

## Results

Six dogs did not reach the learning criterion in the training phase (four in the first day and two dogs in the second day) and eight made more than one mistake in the training trials of the test phase and were therefore excluded from further testing. Therefore, complete data were obtained from 15 of the 29 dogs that were initially recruited. Binomial logistic regression revealed that female dogs (z =  − 1.99, p = 0.047) were significantly more likely to finish the entire experiment successfully than males.

Dogs that completed all four test trials needed on average 18.9 training trials (excluding correction trials) on the first day and 16.9 training trials (excluding correction trials) on the second day to reach the learning criterion.

During test trials, the dogs chose the unconnected stimulus significantly more often than expected by chance (39/60; z = 2.29, p = 0.02; generalized linear mixed model).

During the training trials in the test phase, dogs looked at the positive stimulus 31.4 ± 21.6% of the presentation time, at the negative stimulus 25.4 ± 20.5% of the presentation time, at the presenting experimenter 41.2 ± 25.9% of the presentation time and elsewhere for 2.0 ± 6.5% of the presentation time. During test trials, dogs looked at the unconnected stimulus 30.9 ± 23.5% of the presentation time, at the connected stimulus 29.8 ± 21.6% of the presentation time, at the presenting experimenter 37.8 ± 25.5% of the presentation time and elsewhere for 1.5 ± 4.6% of the presentation time.

The generalized linear mixed model revealed that the longer the dogs looked at the unconnected stimulus during the presentation, the more likely they were to choose it (z = 2.483, p = 0.01). No effect was found for sex (z = 0.155, p = 0.88), number of training trials (z = 0.205, p = 0.84), or the percentage of time spent looking at the connected panel during the presentation (z = -0.700, p = 0.48).

## Discussion

The present study aimed at deepening our knowledge on perceptual biases affecting numerical estimation by dogs. To this purpose, we first trained dogs to choose the larger of two sets of food items. Then, two sets were presented with the same amount of food items (30 vs. 30), but in one array items were interconnected by lines. Dogs significantly selected the array with unconnected pieces of food, showing a relative underestimation of the array composed by interconnected items, as reported in humans^30–32,34–38^. To date, this is the first evidence that a non-human animal species is susceptible to the effect of item connection, on the ability to estimate quantity.

Our study provides one of the few evidence of similarities between humans and dogs in the perception of illusory patterns. As opposed to humans, dogs appear to be not susceptible to the Delboeuf^49,50^, Ponzo^51,52^, and Müller-Lyer illusion^53^; moreover dogs are susceptible to the Ebbinghaus illusion, but with an opposite effect than what observed in humans^49^. All the above-mentioned illusions belong to the category of size^54^ or distortion illusions^55^, but the different susceptibility to such illusions observed in dogs suggests that different mechanisms may be at play in their perception, and that only part of these mechanisms may be shared by dogs and humans.

Possibly, the most informative considerations arise when comparing dogs susceptibility to the connectedness illusions along with their lack of susceptibility to another numerical estimation illusion, the solitaire illusion^47^. It should be premised that both the solitaire and the connectedness illusion are thought to originate from Gestalt, although involving different principles: in the solitaire illusions, the perception of a single Gestalt relies on the principles of both proximity, whereby items tend to be perceived as a whole if close to one another, and good continuation, which entails the grouping of items that are arranged in a straight line^25^. In the connectedness illusion, the principle driving the Gestalt is that of closure, whereby elements forming visual wholes are readily organized into single units^56^. Therefore, one possibility to explain dogs’ susceptibility to connectedness but not the solitaire illusion is that closure may be more effective in eliciting a Gestalt than proximity and good continuation of items, at least in dogs. This, in turn, could be linked to different extent by which dogs and humans prioritize global configurations over local elements in the processing of visual hierarchical stimuli—a phenomenon often referred to as ‘seeing the forest before the trees’; in fact, dogs’ tendency in this regard is weaker, compared to the strong global precedence observed in humans^57,58^. However, it must also be noted that perceptual grouping has opposite effects in terms of quantity estimation in the two illusions: elements forming a visual whole are underestimated in the connectedness illusion, but are overestimated in the solitaire one. It is therefore clear that the different susceptibility of dogs to the two illusions cannot be simply explained by the effectiveness by which they evoke a Gestalt or—in other words—by the ‘strength’ of the illusion, but by the involvement of qualitatively different mechanisms.

Some insight can be found in studies in humans: although non-symbolic numerical abilities are generally believed to be functionally located within humans’ parietal lobe (for a review see^59,60^, recent neuroimaging evidence on people exposed to the connectedness illusion showed that activity in the occipital cortex, especially in area V3, represents a crucial node to transform sensory information into subjective experiences of quantity^61^. Unfortunately, comparable neuroimaging data upon exposure to the solitaire illusion is not available. However, it is likely that connected elements are already processed in V3, an area that is crucial to the identification of unitary shapes^32^, while elements characterized by proximity and good continuation are processed elsewhere in the brain. We do not have any data on neurophysiological correlates of our dogs’ performance, and therefore we can only speculate on the matter. However, the human-like susceptibility to the connectedness illusion shown by our dogs raises the intriguing possibility that stimuli characterized by proximity and good continuation may be processed by different neurocognitive systems than those acting on the closure/connectedness, and that only the latter mechanism may be shared between dogs and humans. Whether in dogs such processing is associated with activity in area V3, as it is in humans, is a hypothesis that should soon be tested.

It is worth noting that the time spent looking at the unconnected items before dogs’ choice was positively correlated with their final choices. The positive correlation between looking behaviour and dogs’ choices has been recently reported also by Miletto Petrazzini et al.^46^ in another food choice task. The fact that dogs looked at the unconnected stimulus and then selected this array indirectly resembles a phenomenon known in cognitive science as “quiet eye”, defined as the final fixation or tracking gaze at a task-relevant location immediately prior to movement^62^. This result might have interesting methodological implications. Recording the stimulus reached by the subjects sometimes is problematic in binary choices as it introduces spatial biases not directly related to animals’ capacity to make relative quantity judgments, such as the spontaneous tendency to turn consistently on one side of the environment (e.g., right) regardless to the position of the most favourable group^46,63,64^. The observation of looking time, without releasing the dogs, may be a reliable measure of dogs’ discriminative abilities in food choice tasks.

Finally, a look at the composition of the sample that reached the end of the experiment made it clear that females had been more successful than males at completing the entire experiment, something which was then assessed, and found significant, by statistical analysis. The results must be taken with caution: the experiment had not been run with the aim of looking for sex differences and the initially recruited sample was unbalanced in this regard (11 males and 18 females). Therefore, the differential performance as a function of sex can be, at least partially, explained by the initial sampling bias. Notwithstanding, the striking difference in performance between the two groups prompts to consider other potentially contributing factor. One possible explanation entails different attentional levels between males and female. Previous studies showed that women outperform men in certain attention tasks^65^ and a recent study found differences in patterns of attention by female and male dogs, in favour of the former^66^. Thus, the possibility that female dogs may show better abilities in maintaining attention across a relatively long task is warranted and encourages more research in the area of sex differences in dog’ attention and cognition.

To conclude, we conducted the first assessment of susceptibility to the connectedness illusion in a non-human animal species. The results also represent one of the very few instances of dogs’ human-like susceptibility to illusions, and the only one among the so-called ‘distortion’ illusions. Further studies are necessary to deepen this issue and form a comprehensive understanding of perceptual mechanisms underlying quantitative judgments in dogs. Such investigation could help us to understand how much humans and dogs share similar cognitive/perceptual mechanisms underlying quantity judgments, a fundamental step for finding proper animal models of non-symbolic quantificational abilities.

## Methods

### Subjects

Dogs were recruited through the Laboratory of Applied Ethology database of volunteers. The criteria for recruitment were that dogs were food motivated, in good health and adults (1 to 7 years old). Overall, 29 pet dogs (18 females, 11 males) were recruited, of which 14 did not complete the entire procedure. The 15 dogs which completed the entire test phase were 12 females and 3 males (mean age ± SD of 3.6 ± 1.8 years). Eight dogs were mixed breeds and seven were purebred from various dog breeds.

### Experimental setting

**Figure 2:**
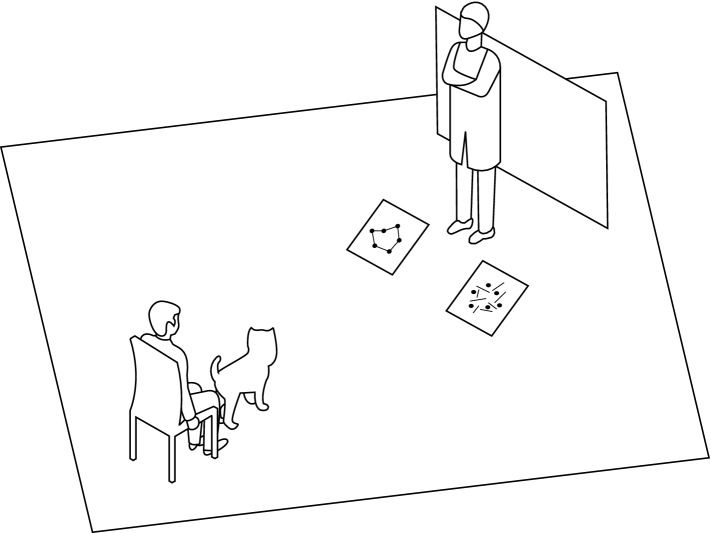
Representation of the experimental setting, illustrating the position of the dog, the owner, the experimenter and the stimuli, during a presentation, before the dog was allowed to choose. Figure not to scale.

Dogs were tested in a quiet room measuring 4.7 × 5.8 m (Fig. 2). The room was equipped with a table behind a 1.3 m tall barrier. Two wooden stands were placed 1 m apart in front of the barrier to hold the panels during the presentation. One meter from the middle point of the stands, a sign was marked on the floor to indicate the position of the dogs’ head during the presentation, and a chair for the owner behind it. Two camcorders (Xacti VPC-WH1, Sanyo, Moriguchi, Japan) were used to record the experiment, one facing the dog and the other facing the panels.

### Stimuli

The stimuli consisted of two panels, with different arrangements of food and non-food elements placed upon them. The panels were square shaped (30 cm × 30 cm × 0.5 cm thickness) and made of black polycarbonate. The food elements were discs cut out from turkey ham slices (1 cm diameter × 1 mm thickness). The non-food elements were thin lines made of white plastic masking tape (2.5 cm × 2.0 mm).

A panel containing 30 discs and 30 lines served as the positive stimulus in the training phase. The simultaneously presented negative stimulus was a panel with 10 discs and 10 lines. On all stimuli the dots were arranged semi-randomly with at least 1 cm distance between individual discs. Approximately half of the lines were in contiguity with the discs on both ends, while the rest were not. All elements were homogeneously spread across the panel and the convex hull for both stimuli was approximately 550 cm^2^. Several panels with different arrangements were used both as positive and negative stimulus.

In the first trials of the training phase (see below) dogs were presented with a negative stimulus where the food elements were replaced with discs made of polymer clay with an identical colour and size to the real food elements. To control for odour cues, a piece of turkey ham was placed on the back of the negative stimulus panel, so to match the overall quantity of ham used in the positive stimulus.

**Figure 3:**
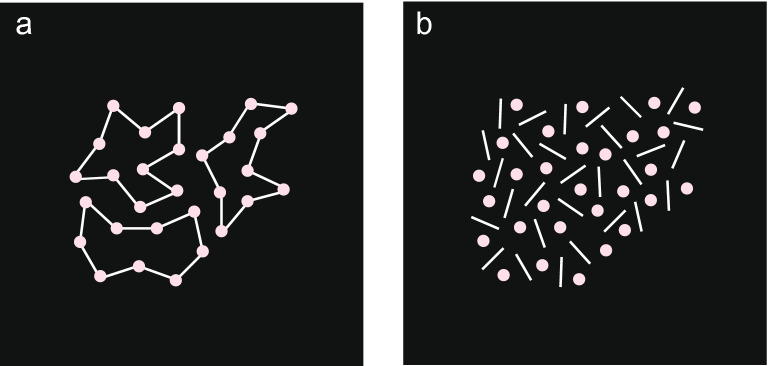
Representation of the test stimulus with unconnected (**a**) and connected (**b**) elements. Food elements are represented by pink dots and inedible lines by white lines.

Two panels, both containing 30 food elements and 30 lines, served as the test stimuli. Discs on both stimuli had an identical spatial arrangement; however, on one stimulus discs were connected by the lines forming three clusters of ten discs each (Fig. 3a) and on the other stimulus, the lines were not in contact with the discs (Fig. 3b). Each dog was presented with four different arrangements of test stimuli.

### Procedure

The experiment was carried out in two different days. The first day started with the dog being brought into the experimental room and let off leash for about 5 min to become acquainted with the area. Meanwhile, the owner was instructed to sit on the chair and keep the dog in between her/his legs in a marked location; owners were instructed to gently hold their dog from the shoulders or from the harness and to look at a fixed position in the wall in front of them.

When owner and dog were in position, an experimenter, who stood between the two stands, presented a single central panel with three food pieces on it and told the owner to release the dog, who was free to reach the panel and eat the food. This preliminary presentation was meant to ascertain that dogs were food-motivated and comfortable in approaching the experimenter to retrieve food. Following this preliminary trial, the actual training phase began. This phase was intended to train dogs to choose the larger of two sets of food items. Although most dogs spontaneously make such choice when presented with food sets of sufficiently different size, this does not seem to apply to the vast majority of dogs (e.g. Lõoke et al.^47^). Therefore, training was necessary to rule out that a choice of the non-connected stimulus in the subsequent test phase was not due to a choice based on quantity.

Each training trial started with the experimenter standing between the stands while a second experimenter behind the barrier passed two panels into her hands to limit as much as possible unnecessary movements. The presenting experimenter placed the panels simultaneously on the stands, then tapped shortly on both panels to attract the dogs’ attention to them. After 5 s the experimenter said “OK” and the dog was released. In the first trials of the phase the panel with food-imitation was used as negative stimulus. If the dog approached it, it was allowed to explore it (e.g. look at, sniff or lick it), while the positive stimulus was quickly removed, so to not allow the dog to eat off it. The panel with food imitation was used until the dog made two correct choices in a row, after which the real-food panel was used as negative stimulus. In these trials, if the dog approached the negative stimulus, both panels were quickly removed before the dog could eat from any of the two, whereas if the dog approached the positive stimulus, it was allowed to eat the food off the panel, to reinforce the choice. After choosing and eventually eating, the dog was then taken back to the initial starting position for the next trial. If the dog had approached the negative stimulus, it was presented with a correction trial (i.e. positive and negative stimuli in the same position as the previous trial) until it chose the positive stimulus. When the dog chose the positive stimulus for five consecutive trials, the training phase was considered successfully completed. If the dog did not reach the learning criterion within 15 trials the dog went out for a 10-min break, before undergoing another training session. The training phase continued until either the dog reached the learning criterion or four training sessions of 15 trials were completed without reaching the learning criterion or the experiment exceeded the time limit of two hours. In the case the dog did not reach the learning criterion in the time limit, it was released from further testing. The position of the stimuli in each trial was pre-determined according to semi-random sequence of 15 trials. Within the sequence, the positive stimulus was presented 7 times on one side, and 8 times on the opposite, and never presented on the same side for more than two trials in a row.

Dogs that reached the learning criterion underwent a second training session two to six days after the first day. This second training meant to ensure the dog was still able to perform the task, before being tested for susceptibility to the illusion. If the dog did not successfully complete this second training phase within the same limits used in the first day, testing was interrupted, and the dog’s data were eliminated from the study. Dogs reaching the learning criterion were presented with the test phase on the same day, after a break of 30 min.

The test phase consisted of four test trials and eight training trials. The procedure was the same as described above, but correction trials were not allowed and only panels with real food elements were used. In test trials, test stimuli were used and dogs were allowed to eat off from the panels regardless of their choice. The trial sequence was semi-randomised, with the constraints that test trials could never being presented as the first trial, two test trials could never being presented one after another and no more than three training trials in a row were presented. The side of the connected and the unconnected stimulus were randomised and counterbalanced across the whole test phase. In case the dog made more than one mistake in the training trials of the test phase, the dog went back to the training phase. If the dog was unable to reach the learning criterion or made two mistakes in the test phase for the second time, it was released from further testing, and its data were not used for analysis.

### Data collection and analyses

The number of trials in the training phase and the dogs’ choices of either stimulus both in the training phase and in the test phase were collected during testing as a binary variable. The same data was later collected from the videos, resulting in 100% agreement.

Behavioural data were collected from videos using the Observer XT software (version 12.5, Noldus, Grœningen, The Netherlands). A continuous sampling method was used to collect data about dogs’ head orientation (looking either at the panel on the right, the panel on the left, the presenting experimenter or elsewhere) during the test trials from the moment the panels became visible to the dog, until the moment in which the dog started moving to make its choice. Dogs’ head orientation data collected by a second independent observer (30% of videos) resulted in a high inter-observer reliability (Pearson’s correlation; looking at the panel on the right: r = 0.97, p < 0.001, looking at the panel on the left: r = 0.97, p < 0.001, looking at the experimenter: r = 0.97, p = 0.004).

A binomial logistic regression model was run to determine if the sex of the dog explained its’ success in completing all the test procedure as hypothesised in previous studies^67,68^. The dependent variable in the model was the dogs’ success in completing the experiment (i.e. dog was presented with all four test trails) and the independent variable was the dog’s sex.

To determine dogs´ susceptibility to the illusion, an intercept-only generalized linear mixed model was run to test the null hypothesis (H0) that dogs’ choices in the test phase were not different from chance level. The subject was included in the model as a random effect.

A generalized linear mixed model was run to determine if dogs’ choices during the test trials was affected by the dog’s sex, the number of training trials to reach the criterion combined for both days, the percentage of time spent looking at the unconnected or the connected panel during the presentation. The subject was included in the model as a random factor.

All statistical analyses were conducted using R, with the statistical significance level set at 0.05. The generalized linear mixed models were fitted using the function glmer of the package lme4^69^.

### Ethics declaration

The study was conducted in accordance with relevant legislation for research involving animal. All experimental protocols were approved by Committee Responsible for Animal Welfare (Organismo Preposto al Benessere Animale, OPBA) of the University of Padua.

Dog owners volunteered to participate in the experiment and permission for involving their dogs was obtained by owners prior to their involvement in the experimental procedures.

## Data Availability

Data are publicly available in the data repository of the University of Padua.
